# Interaction of MRE11 and Clinicopathologic Characteristics in Recurrence of Breast Cancer: Individual and Cumulated Receiver Operating Characteristic Analyses

**DOI:** 10.1155/2017/2563910

**Published:** 2017-01-04

**Authors:** Cheng-Hong Yang, Sin-Hua Moi, Li-Yeh Chuang, Shyng-Shiou F. Yuan, Ming-Feng Hou, Yi-Chen Lee, Hsueh-Wei Chang

**Affiliations:** ^1^Department of Electronic Engineering, National Kaohsiung University of Applied Sciences, Kaohsiung, Taiwan; ^2^Department of Chemical Engineering & Institute of Biotechnology and Chemical Engineering, I-Shou University, Kaohsiung, Taiwan; ^3^Cancer Center, Kaohsiung Medical University Hospital, Kaohsiung Medical University, Kaohsiung, Taiwan; ^4^Translational Research Center, Kaohsiung Medical University Hospital, Kaohsiung Medical University, Kaohsiung, Taiwan; ^5^Institute of Clinical Medicine, Kaohsiung Medical University, Kaohsiung, Taiwan; ^6^Kaohsiung Municipal Hsiao-Kang Hospital, Kaohsiung Medical University, Kaohsiung, Taiwan; ^7^Department of Anatomy, School of Medicine, College of Medicine, Kaohsiung Medical University, Kaohsiung, Taiwan; ^8^Institute of Medical Science and Technology, National Sun Yat-sen University, Kaohsiung, Taiwan; ^9^Department of Biomedical Science and Environmental Biology, Kaohsiung Medical University, Kaohsiung, Taiwan; ^10^Department of Medical Research, Kaohsiung Medical University Hospital, Kaohsiung Medical University, Kaohsiung, Taiwan

## Abstract

The interaction between the meiotic recombination 11 homolog A (MRE11) oncoprotein and breast cancer recurrence status remains unclear. The aim of this study was to assess the interaction between MRE11 and clinicopathologic variables in breast cancer. A dataset for 254 subjects with breast cancer (220 nonrecurrent and 34 recurrent) was used in individual and cumulated receiver operating characteristic (ROC) analyses of MRE11 and 12 clinicopathologic variables for predicting breast cancer recurrence. In individual ROC analysis, the area under curve (AUC) for each predictor of breast cancer recurrence was smaller than 0.7. In cumulated ROC analysis, however, the AUC value for each predictor improved. Ten relevant variables in breast cancer recurrence were used to find the optimal prognostic indicators. The presence of any six of the following ten variables had a high (79%) sensitivity and a high (70%) specificity for predicting breast cancer recurrence: tumor size ≥ 2.4 cm, tumor stage II/III, therapy other than hormone therapy, age ≥ 52 years, MRE11 positive cells > 50%, body mass index ≥ 24, lymph node metastasis, positivity for progesterone receptor, positivity for epidermal growth factor receptor, and negativity for estrogen receptor. In conclusion, this study revealed that these 10 clinicopathologic variables are the minimum discriminators needed for optimal discriminant effectiveness in predicting breast cancer recurrence.

## 1. Introduction

Breast cancer is the most common cancer in women worldwide and is diagnosed in one in three of all women with cancer. Reported risk factors for breast cancer include age, family history, genetic specificity, and lifestyle [1–4]. Local and/or systematic treatments for breast cancer now enable a high survival rate, especially when breast cancer is diagnosed at an early stage [5]. However, breast cancer recurrence or metastasis (i.e., the spread of tumor cells from the original site) can reduce survival time [6].

Prognostic indicators of breast cancer complication and recurrence can be used to predict survival after diagnosis of breast cancer [7]. Estrogen receptor (ER), progesterone receptor (PR), and human epidermal growth factor receptor 2 (HER2) are reportedly accurate and independent prognostic indicators of breast cancer recurrence risk [6], and combining these independent indicators can improve accuracy in predicting recurrence. Representative prognostic indicators can also be used to evaluate the effectiveness of adjuvant therapy and to estimate the risk of tumor recurrence [8].

Our previous study [9] reported the important role of meiotic recombination 11 homolog A (MRE11) in cell proliferation, tumor invasion, and DNA repair in patients with breast cancer. The MRE11 is considered an oncoprotein because it is overexpressed in colorectal cancer [10] and in highly malignant breast cancer [9]. However, no studies have evaluated the use of breast cancer tumor marker MRE11 as a diagnostic or prognostic indicator in breast cancer. Specifically, no studies have evaluated whether MRE11 interacts with clinicopathologic variables associated with breast cancer recurrence.

Receiver operating characteristic (ROC) analysis is widely used method of evaluating the performance of a diagnostic test according to a continuous spectrum of results [11, 12]. By graphically depicting the quantitative analysis results, the ROC curve reveals the discriminant thresholds based on the probability of positive results (sensitivity against 1 − specificity) in individual subjects [13]. The area under the curve (AUC) is a measure of the overall accuracy of the dichotomous methods of the measurements. Recently, ROC has been used as a tool for comparing the accuracy of various models for predicting cancer diagnosis, prognosis, and survival [14–20].

This study developed a scoring system based on ROC analysis to identify patient characteristics associated with susceptibility to breast cancer recurrence. Thus, aims of this study were (i) to assess breast cancer recurrence based on MRE11 expression and clinicopathologic characteristics and (ii) to identify the patient characteristics that are risk factors for breast cancer recurrence.

## 2. Methods

### 2.1. Study Participants

After obtaining IRB approval, this study enrolled 254 female breast cancer patients who had received surgical treatment for pathology-confirmed invasive ductal carcinoma at the Department of Surgery, Kaohsiung Medical University Hospital, during 2006–2010. Informed consent was obtained from all patients. The ethics statement, laboratory procedures, and other study procedures were identical to those in our previous study [9]. The dataset for all clinicopathological variables is available online at https://wp.kmu.edu.tw/changhw/files/2015/10/ROC_MRE11_DATASET.xlsx.

### 2.2. Criteria for Breast Cancer Recurrence

Patients with and without breast cancer recurrence were classified into a recurrence group and a nonrecurrence group, respectively. In the recurrence group, breast cancer recurrence was defined as a local/regional recurrence with or without distant metastasis diagnosed according to symptoms observed in clinical examination, pathology study, or imaging study. Patients who remained disease-free for 60 months after diagnosis or who were disease-free at the end of the follow-up period were classified into the nonrecurrence group.

### 2.3. Dichotomous Results of ROC Analysis for Each Clinicopathologic Variable

The 13 clinicopathologic variables included in the ROC analysis included MRE11 positive cells (%), tumor stage, tumor grade, age, body mass index (BMI), tumor size, lymph node (LN) metastasis, estrogen receptor (ER) status, progesterone receptor (PR) status, human epidermal growth factor receptor 2 (HER2) status, radiotherapy (RT), chemotherapy (CT), and hormone therapy (HT). First, the clinicopathologic variables were dichotomized by ROC curve analysis. The ROC curve is a graphical plot of the true positive rate (sensitivity) against the false positive rate (1 − specificity) at various threshold settings. Each cut-off point estimated by ROC analysis indicates the distinguishing characteristic of each clinicopathologic variable used to classify participants into the recurrence group. The area under the ROC curve (AUC) is used to calculate the accuracy of dichotomous results.

### 2.4. AUC of Cumulated ROC Analysis

A cumulated ROC analysis was performed to detect the combined effects of the clinicopathologic variables used to predict recurrence. Variables that had a strong association with recurrence (i.e., values larger than the cut-off point in AUC from the individually dichotomous results) received a score of 1. The remaining variables received a score of 0 (i.e., values less than the cut-off point in AUC from the individually dichotomous results). The indicators were then ranked by cumulated AUC results for individually dichotomous results. Positive changes in the cumulated AUC values for these variables were tracked until the addition of other variables no longer increased the AUC values. Accordingly, clinicopathologic variables that contributed to positive changes in AUC were selected for further analysis.

### 2.5. Cut-Off Point for Cumulated Scoring System

The cumulated scoring system was then used to rank the clinicopathologic variables as breast cancer predictors. The cumulated score for each subject was obtained by adding the risk factors to the recurrence score (1 or 0). The correctly classified rate for each possible cut-off point within the range of cumulated scores was dependent on the number of clinicopathologic variables selected for ROC analysis. For example, if “*n*” variables were selected according to a positive change in AUC value in the previous step, cumulated scores ranging from 0 to “*n*” were generated. The last step was calculating the specificity, sensitivity, and correctly classified rate for each cut-off point in the cumulated score range.

### 2.6. Risk Relationship of the Selected Variables in the Cumulated Scoring System

For each subject, the cumulated score represented the total number of clinicopathologic variables that were breast cancer risk factors. For instance, a score of 3 was interpreted as the presence of three risk factors that were more relevant to breast cancer recurrence compared to the selected clinicopathologic variables.

### 2.7. Statistical Analyses

Differences in the distributions of clinicopathologic variables between the recurrence and the nonrecurrence groups were estimated by frequency tables and the *χ*^2^ test. The AUC represents the accuracy of the dichotomous results for a single clinicopathologic variable for predicting breast cancer recurrence. The dichotomous results with high AUC values were considered better predictors of breast cancer recurrence.

In cumulated ROC analysis, the likelihood ratio was used to assess recurrence status in subjects with different cumulated scores. The likelihood ratio for a positive test result [LR+: sensitivity/(1 − specificity)] represents the ratio of the probability of a positive test in the recurrence subjects to the probability of a positive test in the nonrecurrence subjects. Comparatively, the likelihood ratio for a negative test [LR−: (1 − sensitivity)/specificity] result represents the ratio of the probability of a negative test in the recurrence subjects to the probability of a negative test in the nonrecurrence subjects. All statistical analyses were performed by STATA version 11.0.

## 3. Results

### 3.1. Clinicopathologic Characteristics and Recurrence of Breast Cancer

During the 5-year follow-up period, recurrence developed in 34 (13.39%) of the 254 breast cancer patients.  Table 1 compares clinicopathological variables between the recurrence and the nonrecurrence groups. Compared to the nonrecurrence group, the recurrence group had significantly larger proportions of patients with MRE11 positive cells > 50% (85.29%), breast cancer stage II/III (94.12%), age ≥ 52 years (67.65%), tumor size ≥ 2.4 cm (67.65%), LN metastasis (58.82%), negative expression of ER (58.82%), negative expression of PR (64.71%), triple negative breast cancer (35.29%), RT (79.41%), and no-HT treatment (61.76%).

### 3.2. AUC for Clinicopathologic Variables for Breast Cancer Recurrence

Table 2 shows the AUCs obtained when these 13 variables were considered in estimates of recurrence risk. The clinical criteria for high and low risk of breast cancer recurrence were identical to those used in our previous study [9]. Notably, although the dichotomized tumor size yielded the highest AUC value (0.679 with sensitivity of 0.677 and specificity of 0.682), it did not meet the criterion of AUC ≥ 0.7 [21] for classification of recurrence.

### 3.3. Cumulated ROC Analysis of Breast Cancer Recurrence

No single dichotomized variable showed satisfactory performance in predicting breast cancer recurrence (defined as AUC < 0.700). Therefore, this study developed an improved scoring system that considered the combined effects of these variables.  Table 3 shows the ROC analysis results obtained for the developed scoring system with cumulated top-ranked predictors. The scoring system obtained a good AUC value (0.806) when 6 dichotomized variables (tumor size, tumor stage, ER, HT, LN metastasis, and age) were used. The AUC values showed further positive changes (range, 0.806 to 0.821) when the scoring system consisted of the cumulated top tenth rank of dichotomized variables (tumor size, stage, ER, HT, LN metastasis, age, PR, MRE11 positive cells, BMI, and HER2). When the number of variables exceeded ten cumulated top-ranked variables, however, the AUC value slightly decreased. That is, the scoring system obtained the best AUC values when ten dichotomized variables were used.

### 3.4. Cut-Off Point for Cumulated Scoring System

Hence, the performance of possible cut-off points ranging from score 0 to 10 were compared in the scoring system.  Table 4 compares the results. For predicting recurrence, a cut-off point of 0 had a sensitivity of 100% (all recurrence patients correctly classified) but had a specificity of 0% (no recurrence patients correctly classified). In contrast, a cut-off point of 100% had a specificity of 10 (all nonrecurrence subjects correctly classified) but a sensitivity of 0 (no recurrence patients correctly classified). The cut-off point for 6 dichotomized variables had the highest sensitivity and the highest specificity. This cut-off point correctly classified 71.3% with the best combination of sensitivity (0.794) and specificity (0.700; LR+ 2.647 and LR− 0.294).

### 3.5. Risk Relationships of the Selected Variables in the Cumulated Scoring System

Table 5 lists the clinicopathologic variables that contributed the five largest possible changes in AUC values (0.799 to 0.821) in Table 3. The scores for 10 dichotomized variables were then computed into these five clinicopathologic variables as indicated in Table 5. In the 254 patients analyzed, scores were ≤ 5 in most (63.39%; 161) patients. In the 161 patients with scores of ≤ 5, most (63.98%, 103) patients were HER2 negative. The score was 6 in 16.93% (43/254) of the patients. In patients with a score of 6, most (74.42%; 32/43) had BMI ≥ 24. The score was 7 in 9.45% (24/254) of the patients. In patients with a score of 7, all (100.00%) had BMI ≥ 24. The score was 8 in 6.69% (17/254) patients. In patients with a score of 8, 88.24% (15) had BMI ≥ 24. The score was 9 in 3.15% (8/254) patients. All patients who had a score of 9 were negative for PR expression and had a BMI ≥ 24. Only 0.39% (1/254) patients had a score of 10, that is, high values for all five clinicopathologic variables that were risk factors for breast cancer recurrence.

## 4. Discussion

Conventional statistical methods used to estimate probability of breast cancer recurrence include logistic regression, Cox-proportional hazard regression model, Kaplan-Meier estimator, and log-rank test [22]. Our previous work using similar statistical methods revealed that MRE11 is associated with breast cancer malignancy [9]. However, possible interactions between MRE11 and clinicopathologic variables for breast cancer recurrence have not been reported in the literature.

An ROC analysis is a simple and powerful approach to discriminant analysis. In this study, dichotomization of clinicopathologic variables by AUC enabled quick and easy differentiation of variables associated with high and low recurrence risk. However, variations in breast cancer recurrence are rarely affected by a single factor. Similarly, we found that the contribution of each variable may be too weak (<0.7) in terms of AUC.

A recent study developed a cumulated ROC analysis strategy for assessing outcomes of orthodontic surgery [23] and for diagnosing metastasis in breast cancer [24] and other cancer types [25, 26]. In our study, a similar scoring system was applied in multivariate cumulated ROC analysis to evaluate diagnostic indicators of breast cancer recurrence.  Table 3 shows that 10 variables were the minimum number of discriminators required to obtain the optimum discriminant effectiveness.  Table 4 further shows that a cut-off point of 6 had the best combination of sensitivity and specificity for predicting breast cancer recurrence. These results suggest that the cumulated ROC analysis strategy also improves accuracy in predicting breast cancer recurrence.

Moreover, these data suggest that both gene-environment and environment-environment interactions have important roles in predicting recurrence when clinicopathologic variables are regarded as the environmental factors. Similar interactions between these variables have been reported. For example, ER and HER2 are reportedly both interdependent and independent prognostic indicators of breast cancer recurrence [27]. The Nottingham prognostic index considers tumor size, lymph node metastasis, and tumor grade to obtain an estimate of recurrence risk in patients with breast cancer [28, 29]. In contrast, the breast cancer severity score (BCSS) is a prognostic scoring system based on tumor size, number of metastatic lymph nodes, and HER2 status [30]. The BCSS may be the best predictor of both overall survival and disease-free survival. A common feature of these prognostic scoring systems is that all of the breast cancer characteristics mentioned in the current study are considered simultaneously.

Comparisons of these variables further showed that most subjects with a high risk of breast cancer recurrence had a high BMI (Table 5), which is consistent with reports that high BMI is associated with aggressive tumor characteristics in premenopausal [31] and postmenopausal women [32]. Another randomized trial showed that lymph node metastasis, ER-negativity, and HER2 negativity are associated with breast cancer risk and prognosis [33]. Breast cancer patients with the triple negative subtype (i.e., negativity for ER, PR, and HER2) have a high risk of disease progression [34]. Supplementary Table  1 (in Supplementary Material available online at https://doi.org/10.1155/2017/2563910) shows that MRE11 expression did not significantly differ among ER-positivity, ER-negativity, PR-positivity, or PR-negativity in the current study. Supplementary Table  2 further shows that BMI level did not significantly differ among ER-positivity, ER-negativity, PR-positivity, PR-negativity, HER2-positivity, or HER2-negativity.

Some limitations of this study should be noted. First, our previous work [9] suggested a survival analysis of the current dataset using Cox-proportional hazard model, Kaplan-Meier curve, and log-rank test. However, survival was not considered in the follow-up analyses of subjects in the current study. Another limitation is that, although the cumulated ROC analysis revealed potential joint effects of the selected clinicopathologic variables, the complex interactions of all possible combinations of clinicopathologic variables were not analyzed because the cumulated ROC was initially based on the highest ranked AUC for a single variable. In subsequent analyses, the variable with the lowest AUC was not considered. Moreover, this study did not validate the signature identified by ROC analysis in a testing dataset. Therefore, the use of a testing dataset and intelligent computational algorithms [35–44] is warranted in future studies of the complex high-order interactions between these clinicopathologic variables and breast cancer recurrence.

## 5. Conclusions

This study evaluated the contributions of 13 clinicopathologic variables in predicting breast cancer recurrence. In individual ROC analysis, each variable had a weak AUC for predicting breast cancer recurrence. In cumulative ROC analysis, however, each variable had an improved AUC. Finally, this study revealed that 10 clinicopathologic variables is the minimum number of discriminators needed for optimum accuracy in predicting breast cancer recurrence by discriminant analysis.

## Supplementary Material

Supplementary Table 1. The expression range of MRE11 in the different groups of breast cancer patients. Supplementary Table 2. The distribution of BMI level in the different groups of ER, PR and HER2 breast cancer patients.

## Figures and Tables

**Table 1:** Clinicopathologic characteristics of breast cancer patients in recurrence status^*∗*1^.

Variable	No recurrence (*n* = 220)		Recurrence (*n* = 34)		*P*
	*N*	%	*N*	%	
MRE11 positive cells					0.030
≤50%	73	33.18	5	14.71	
>50%	147	66.82	29	85.29	
Stage					<0.001
I	88	40.00	2	5.88	
II, III	132	60.00	32	94.12	
Grade					0.543
1, 2	166	75.45	24	70.59	
3	54	24.55	10	29.41	
Age					0.007
<52 yrs	126	57.27	11	32.35	
≥52 yrs	94	42.73	23	67.65	
BMI (kg/m^2^)					0.151
<24	126	57.27	15	44.12	
≥24	94	42.73	19	55.88	
Tumor size (cm)					<0.001
<2.4 cm	150	68.18	11	32.35	
≥2.4 cm	70	31.82	23	67.65	
LN metastasis					0.003
Negative	149	67.73	14	41.18	
Positive	71	32.27	20	58.82	
ER					<0.001
Negative	63	28.64	20	58.82	
Positive	157	71.36	14	41.18	
PR					0.009
Negative	90	40.91	22	64.71	
Positive	130	59.09	12	35.29	
HER2 status					0.430
Negative	140	63.64	24	70.59	
Positive	80	36.36	10	29.41	
Triple negative					0.002
No	190	86.36	22	64.71	
Yes	30	13.64	12	35.29	
RT					0.021
No	91	41.36	7	20.59	
Yes	129	58.64	27	79.41	
CT					0.866
No	30	13.64	5	14.71	
Yes	190	86.36	29	85.29	
HT					0.001
No	72	32.73	21	61.76	
Yes	148	67.27	13	38.24	

^*∗*1^Dataset was retrieved from our previous study [9]. MRE11: meiotic recombination 11; BMI: body mass index; LN: lymph node; ER: estrogen receptor; PR: progesterone receptor; HER2: human epidermal growth factor receptor 2; RT: radiotherapy; CT: chemotherapy; HT: hormone therapy.

**Table 2:** AUC of clinicopathologic characteristics for recurrence status^*∗*1^.

Variable	AUC	High risk	Low risk	Sensitivity	Specificity
Tumor size (cm)	0.679	≥2.4 cm	<2.4 cm	0.677	0.682
Stage	0.671	II, III	I	0.941	0.400
ER	0.651	Negative	Positive	0.588	0.714
HT	0.645	No	Yes	0.618	0.673
LN metastasis	0.633	Positive	Negative	0.588	0.677
Age	0.625	≥52 yrs	<52 yrs	0.677	0.573
PR	0.619	negative	positive	0.647	0.591
MRE11 positive cells	0.592	>50%	≤50%	0.853	0.332
BMI	0.566	≥24	<24	0.559	0.573
HER2 status	0.535	Negative	Positive	0.706	0.364
Grade	0.524	3	1, 2	0.294	0.755
CT	0.505	No	Yes	0.147	0.864
RT	0.396	No	Yes	0.206	0.586

^*∗*1^Data for high/low risks of breast cancer recurrence were retrieved from our previous study [9]. AUC: area under receiver operating characteristic; ER: estrogen receptor; HT: hormone therapy; LN: lymph node; PR: progesterone receptor; MRE11: meiotic recombination 11; BMI: body mass index; HER2: human epidermal growth factor receptor 2; CT: chemotherapy; RT: radiotherapy.

**Table 3:** Cumulated top-ranked prediction results using ROC analysis^*∗*1^.

Cumulated top-ranked variables	Variables	AUC
2	Tumor size and stage	0.724
3	Above variables plus ER	0.771
4	Above variables plus HT	0.765
5	Above variables plus LN metastasis	0.790
6	Above variables plus age	0.806
7	Above variables plus PR	0.800
8	Above variables plus MRE11 positive cells	0.799
9	Above variables plus BMI	0.810
10	Above variables plus HER2	0.821
11	Above variables plus grade	0.806
12	Above variables plus CT	0.799
13	Above variables plus RT	0.774

^*∗*1^Dataset and high/low risks of breast cancer recurrence were retrieved from our previous study [9]. ER: estrogen receptor; HT: hormone therapy; LN: lymph node; PR: progesterone receptor; BMI: body mass index; CT: chemotherapy; RT: radiotherapy.

**Table 4:** Cut-off point identified by ROC analysis^*∗*1^.

Number of dichotomized variables^*∗*2^	Sensitivity	Specificity	Sensitivity + specificity	Correctly classified	LR+	LR−
0	1.000	0.000	1.000	0.134	1.000	—
1	1.000	0.027	1.027	0.158	1.028	0.000
2	1.000	0.073	1.073	0.197	1.078	0.000
3	1.000	0.182	1.182	0.291	1.222	0.000
4	0.941	0.332	1.273	0.413	1.409	0.177
5	0.912	0.468	1.380	0.528	1.714	0.189
6	0.794	0.700	1.494	0.713	2.647	0.294
7	0.618	0.868	1.486	0.835	4.686	0.440
8	0.382	0.941	1.323	0.866	6.471	0.656
9	0.177	0.986	1.163	0.878	12.941	0.835
10	0.029	1.000	1.029	0.870	—	0.971

LR+: likelihood ratio for a positive test result; LR−: likelihood ratio for a negative test result.

^*∗*1^Dataset was retrieved from our previous study [9].

^*∗*2^The number of dichotomized variables was the cumulated effects of the various clinicopathologic variables from Table 3, including tumor size, stage, ER, HT, LN metastasis, age, PR, BMI, MRE11 positive cells, and HER2.

**Table 5:** Risk relationship of scores with selected variables^*∗*1^.

Score^*∗*2^	Total	Age ≥ 52 yrs (*n* = 117)		PR negative (*n* = 112)		MRE11 positive cells > 50% (*n* = 113)		BMI ≥ 24 (*n* = 176)		HER2 negative (*n* = 164)	
	*N*	*N*	%	*N*	%	*N*	%	*N*	%	*N*	%
≤5	161	51	31.68	47	29.19	61	37.89	96	59.63	103	63.98
6	43	27	62.79	27	62.79	20	46.51	32	74.42	27	62.79
7	24	18	75.00	16	66.67	13	54.17	24	100.00	14	58.33
8	17	13	76.47	13	76.47	12	70.59	15	88.24	13	76.47
9	8	7	87.50	8	100.00	6	75.00	8	100.00	6	75.00
10	1	1	100.00	1	100.00	1	100.00	1	100.00	1	100.00

PR: progesterone receptor; MRE11: meiotic recombination 11; BMI: body mass index; HER2: human epidermal growth factor receptor 2.

^*∗*1^Dataset was retrieved from our previous study (*n* = 254) [9].

^*∗*2^Cumulated score representing the number of risk properties of the selected clinicopathologic variables in the subjects. The selected clinicopathologic variables included tumor size, tumor stage, ER, HT, LN metastasis, age, PR, BMI, MRE11 positive cells, and HER2.
