# Supplementary material for: Interaction of MRE11 and Clinicopathologic Characteristics in Recurrence of Breast Cancer: Individual and Cumulated Receiver Operating Characteristic Analyses
Source: Biomed Res Int. 2017 Jan 4;2017:2563910. doi: 10.1155/2017/2563910 (PMC5241446; doi:10.1155/2017/2563910)
Supplement: Supplementary file 1 — Supplementary Table 1. The expression range of MRE11 in the different groups of breast cancer patients. Supplementary Table 2. The distribution of BMI level in the different groups of ER, PR and HER2 breast cancer patients. [file 2563910.f1.docx]

| **Supplementary Table 1**. The expression range of MRE11 in the different groups of breast cancer patients | | | | | | |
| --- | --- | --- | --- | --- | --- | --- |
| Variable |  | Mean | SD | Range |  | *P* |
| ER |  |  |  |  |  | 0.596 |
| negative |  | 63.49 | 23.47 | 10-90 |  |  |
| positive |  | 65.06 | 21.34 | 10-90 |  |  |
| PR |  |  |  |  |  | 0.778 |
| negative |  | 64.11 | 23.24 | 10-90 |  |  |
| positive |  | 64.89 | 21.09 | 10-90 |  |  |

| **Supplementary** **Table 2**. The distribution of BMI level in the different groups of ER, PR and HER2 breast cancer patients | | | | | | | | |
| --- | --- | --- | --- | --- | --- | --- | --- | --- |
| Variable |  | BMI (kg/m^2^) < 24 | |  | BMI (kg/m^2^) ≥ 2.4 cm | |  | *P* |
|  |  | n | % |  | n | % |  |  |
| ER |  |  |  |  |  |  |  | 0.984 |
| negative |  | 46 | 32.62 |  | 37 | 32.74 |  |  |
| positive |  | 95 | 67.38 |  | 76 | 67.26 |  |  |
| PR |  |  |  |  |  |  |  | 0.642 |
| negative |  | 64 | 45.39 |  | 48 | 42.48 |  |  |
| positive |  | 77 | 54.61 |  | 65 | 57.52 |  |  |
| HER2 status |  |  |  |  |  |  |  | 0.784 |
| negative |  | 90 | 63.83 |  | 74 | 65.49 |  |  |
| positive |  | 51 | 36.17 |  | 39 | 34.51 |  |  |
